# INFECTIOUS DISEASE: Digging into Seaside Microbial Exposures

**DOI:** 10.1289/ehp.117-a392a

**Published:** 2009-09

**Authors:** Angela Spivey

**Affiliations:** **Angela Spivey** writes from North Carolina about science, medicine, and higher education. She has written for *EHP* since 2001

Beachgoers enjoying the last days of summer might wish to take note: epidemiologist Chris Heaney of the University of North Carolina at Chapel Hill and colleagues from the U.S. Environmental Protection Agency have found that digging in sand and, to a greater extent, being buried in sand are associated with an increased risk of diarrhea and gastrointestinal illness. “The overall incidence of these cases we’re observing is quite low—less than ten percent,” Heaney says. “But such large numbers of people in the country and the world engage in sand contact activities that, even with a very modest relative risk, you could see substantial disease burden.”

As they report in the 15 July 2009 *American Journal of Epidemiology*, Heaney and colleagues analyzed data gathered from 26,609 participants in 2003–2005 and 2007 as part of a joint effort of the Environmental Protection Agency and the Centers for Disease Control and Prevention. Visitors to 7 freshwater and marine beaches in Michigan, Indiana, Ohio, Mississippi, Alabama, and Rhode Island were interviewed at the beach about their activities there. Then, 10–12 days later, an adult from each family participated in a telephone interview about the incidence of sickness since leaving the beach, including diarrhea alone and a broader group of “gastrointestinal illness” symptoms including diarrhea, vomiting, and nausea.

Of the people who dug in the sand, 6% reported diarrhea, whereas among those who didn’t dig in sand, the incidence of diarrhea was 4%. After accounting for other factors that might cause illness, that amounted to a 20% increase in risk for those exposed, Heaney says. Among people who reported being buried in the sand, 9% reported gastrointestinal illness. Among those who weren’t buried, 7% had gastrointestinal illness. That amounted to a 23% risk increase after accounting for other factors.

Heaney’s study comes even as amendments to the Federal Water Pollution Control Act to strengthen pathogen monitoring in beach water wind their way through Congress. More than a decade of research has shown that sand at marine and freshwater beaches throughout the United States contains high levels of so-called fecal indicator bacteria. These bacteria, which include *Escherichia coli* and enterococci, are considered markers of sewage contamination and other non–point sources of fecal waste. If found at sufficient levels in water, their presence can result in waterway closures. But current practices don’t take into account their presence in sand.

“[The Heaney study] is the first step showing that we need to look more closely at the risks of exposure to sand at beaches,” says Alexandria Boehm, an environmental engineer at Stanford University. “A year ago, scientists may have thought there are no risks associated with exposure to *Enterococcus* or *E. coli* from beach sand. Then a paper like this comes along, and you have to pause and say, ‘Well, maybe there is a risk.’”

Some studies have suggested that sources of fecal indicator bacteria in sand and water may have little to do with sewage pollution, says Richard Whitman, an ecologist and branch chief at the U.S. Geological Survey Great Lakes Science Center. These and other microbes can come from bird feces, and sand itself makes a good breeding ground for such organisms, studies have shown. “Heaney’s study helps demonstrate that [ensuring beach safety is] far more complex than ‘sewage outfall equals a beachfront closure.’ We need to look at the whole ‘beachshed’ to understand health and pathogen implications,” Whitman says.

Now scientists must explore the possible routes of exposure behind the association between sand and illness. For instance, Heaney is studying whether high levels of fecal indicator bacteria in sand correspond to high incidence of illness. And Whitman reported in volume 7, issue 4 (2009) of the *Journal of Water and Health* that bacteria and viruses from sand transferred readily to hands but that rinsing hands with water removed a large percentage of them.

In the meantime, Heaney’s advice: after playing on the beach, be sure to wash your hands. And don’t swallow the sand!

